# A giant of physics—in memory of Chen-Ning Yang

**DOI:** 10.1093/nsr/nwaf453

**Published:** 2025-10-21

**Authors:** Fu-Chun Zhang, Qi-Kun Xue

**Affiliations:** University of Chinese Academy of Sciences, China; Southern University of Science and Technology, China; Tsinghua University, China

## Abstract

Chen-Ning Yang, the most renowned Chinese scientist of all time and one of the most influential physicists in the second half of twentieth century, passed away in Beijing on October 18, 2025 at the age of 103. Dr. Yang had made invaluable contributions to China’s science and education. His legacy will last forever.

Chen-Ning Yang (杨振宁), professor at Tsinghua University, the most renowned Chinese scientist of all time and one of the most influential physicists in the second half of the twentieth century, passed away in Beijing on 18 October 2025, at the age of 103. Dr. Yang had made monumental contributions to China’s science and education. His legacy will last forever.

Dr. Yang’s seminal contributions to physics cover high-energy physics, statistical mechanics, condensed-matter physics and mathematics physics. Most notably, he shared the Nobel Prize with Dr. Tsung-Dao Lee (李政道) in 1957 for their proposal of ‘parity non-conservation in weak interactions’, which challenged the traditional belief in the symmetry of nature’s law within the physics community. Their theory was soon experimentally confirmed by Dr. Chien-Shiung Wu (吴健雄) and colleagues, marking a watershed moment in physics. This discovery not only reshaped humanity’s understanding of natural laws, but also exemplified the spirit of ‘daring to question authority and seeking truth through practice’ in scientific exploration.

The ‘Yang-Mills gauge theory’ that Dr. Yang proposed in 1954, considered by himself to be even more profound than parity non-conservation, is generally regarded as a central pillar of physics, on a par with Maxwell’s equations and Einstein’s theory of general relativity, and laid the foundation for the subsequent development of the electroweak unification theory and quantum chromodynamics, the Standard Model of particle physics and the Grand Unified Theory.

Beyond his scientific achievements, Dr. Yang’s contributions to China also transcended time and space. In 1971, he became the first renowned scientist to visit China, serving as an ‘icebreaker’ in China–US academic exchanges and inspiring a wave of overseas Chinese scholars to return to their homeland. In the decades that followed, he raised funds to establish the Committee on Educational Exchange with China, sponsoring nearly 100 Chinese scholars for advanced studies in the US. In the late 1990s, Dr. Yang founded the Institute for Advanced Study at Tsinghua University, which has become a center of theoretical sciences in China and in the world. In 2003, he moved permanently to the Tsinghua campus, naming his residence ‘Root-Returning Pavilion’. After his return to China, Dr. Yang greatly promoted physics in China and especially in Tsinghua University. He nurtured many generations of Chinese scientists with his academic motto: ‘Better to be clumsy than crafty; better to be plain than ornate.’ He once remarked that his most important contribution in life might have been ‘helping to boost Chinese people’s self-confidence’—a legacy that has enriched the nation’s scientific soil.

Dr. Yang had great taste in science, including the fields on which he did not work directly. Soon after the discovery of the quantum Hall effect in 1980, Yang realized its significance because of its relationship with fundamental constants of nature and suggested that his associates should pay attention to the topics. Likewise, Yang was one of the first to immediately recognize the importance of the experimental discovery of the quantum anomalous Hall effect at Tsinghua University in 2013, which has by now been awarded a number of prestigious national and international prizes.

Dr. Yang was both a master who pushed the boundaries of science and a devoted son whose heart remained with his homeland. He not only inherited the essence of Western science, but also carried forward the legacy of Chinese culture. His wisdom and sentiment, bridging East and West, will continue to inspire Chinese scientists and technologists of many generations to come.

Although Dr. Chen-Ning Yang has left us, the theoretical treasures and spiritual heritage he left behind will forever light the path of human exploration and guide Chinese science toward an even broader future. Chen-Ning Yang, a true giant of physics, will be deeply missed.

